# Molecular Identification and Phenotypic Antimicrobial Resistance of *Acinetobacter* spp. from an Equine University Clinic in Germany

**DOI:** 10.3390/antibiotics15060558

**Published:** 2026-05-30

**Authors:** Sabita Diana Stöckle, Anais Sauerwein, Elisabeth Mueller, Heidrun Gehlen

**Affiliations:** 1 Equine Clinic: Surgery and Radiology, Freie Universität Berlin, Oertzenweg 19b, 14163 Berlin, Germany; 2 Laboklin GmbH & Co. KG, Steubenstraße 4, 97688 Bad Kissingen, Germany

**Keywords:** *Acinetobacter* spp., environmental sampling, ESKAPE pathogens, hygiene, wound infections

## Abstract

Background: While equine patient-mediated introduction of A. baumannii into hospital settings has been documented, its environmental dissemination and the risk of hospital-acquired surgical site infection remain poorly understood. Objective: Therefore, this descriptive observational study examined (a) the environmental distribution of *Acinetobacter* spp. in an equine university hospital, (b) the impact of the implementation of new hygiene protocols, (c) the specification of resistance patterns, and (d) the evaluation of the presence of *Acinetobacter* spp. in hospital-acquired wound infections. Methods: During three sampling periods, environmental samples of the stables, the treatment, and surgery areas were collected before and after cleaning and disinfection. After sampling period 1 (December 2021), the cleaning routines were optimized by reviewing the cleaning and disinfection process, as well as including further surfaces in the cleaning schedule for January 2022). This was followed by a second (February 2022) and a third (June 2022) sampling period. During sampling periods 1 and 2, 76 surfaces were sampled; in sampling period 3, only 21 critical surfaces were examined. Samples were cultured on selective agar plates and incubated at 37 °C, with bacterial growth evaluated after 24–48 h. Wound swabs were enriched in broth before culturing. Bacteria were identified using MALDI-TOF mass spectrometry. During the first sampling period, antibiotic susceptibility testing was performed using broth microdilution according to CLSI-vet standards. Results: During each sampling period, *Acinetobacter* spp. was detected in at least one sample in each of the different areas; however, there was a reduced detection rate from sampling period 1 throughout sampling period 3. The isolates were highly resistant against beta-lactam and macrolide antibiotics but mostly sensitive to fluroquinolones (enrofloxacin, 2.2% resistance; marbofloxacin, 0.0% resistance), aminoglycosides (gentamicin, 6.5% resistance; kanamycin, 8.7% resistance), and tetracyclines. *Acinetobacter* spp. was not detected in surgical site infections. Conclusions: Environmental persistence of *Acinetobacter* spp. in an equine clinical setting does not necessarily translate into surgical site infections. Through prudent antibiotic use, the antibiotic susceptibility of the isolates may be perceived.

## 1. Introduction

Hospital-acquired or nosocomial infections are not only an emerging threat concerning human medicine—increased reports of nosocomial infections, especially in large animal hospitals, are a major health concern [1,2,3,4,5,6,7], and pathogens such as Methicillin-resistant *Staphylococcus aureus* (MRSA) may exert zoonotic potential [1].

*Acinetobacter* spp. poses a significant challenge, since they may be part of the human skin and pharyngeal flora [8,9,10,11] and may be persistent in a hospital environment [12]. Furthermore, animals can serve as a potential reservoir for multidrug-resistant (MDR) *Acinetobacter* spp. [13,14,15].

In humans, *Acinetobacter* spp. is implicated in bacteremia, pulmonary infections, meningitis, diarrheal illness, and problematic nosocomial outbreaks, which are associated with mortality rates ranging from 20% to 60% [12]. Also, in small animals, a variety of infections with *Acinetobacter* spp. have been reported, including urinary tract, wound, and skin infections [16,17,18]. In horses, in addition to colonization of the nostrils and the gastrointestinal tract [14,18], detection of *Acinetobacter* spp., mostly *A. baumannii*, was reported from intravenous catheter tips, including those showing signs of thrombophlebitis [13,19], from tracheal washes from horses with respiratory diseases or “poor performance” [20] and foals with bronchopneumonia [21], as well as in the blood culture of a septic foal [22].

Particularly in equine clinics, challenging conditions prevail due to species-specific factors (animal size, bedding in stalls, feed, and stable ventilation). These species-specific factors complicate the development of targeted hygiene measurements, which adds up to difficulties in preventing nosocomial infections [23]. 

Previous studies focusing on injured horses or horses presenting for colic surgery admitted to our clinic identified a 0.9–2.9% *A. baumannii* carrier rate (nasal vestibule, fecal samples) [14,24,25]; its environmental persistence and relevance as a nosocomial pathogen have not yet been assessed in our clinic.

Therefore, the objective of the study was to (a) determine the environmental distribution of *Acinetobacter* spp. in our equine university hospital, (b) to examine the impact of the implementation of new hygiene protocols on the environmental distribution of *Acinetobacter* spp., (c) to specify the resistance patterns of *Acinetobacter* spp. in an equine university hospital, and (d) to evaluate the presence of *Acinetobacter* spp. in hospital-acquired wound infections.

## 2. Material and Methods

### 2.1. Surface Samples

For this descriptive observational study, surface samples for microbiological examination were collected from surface areas of different parts of the clinic. Surfaces were selected based on a risk assessment and on clinical criteria such as frequent contact with hands and/or patients, proximity to patients, and relevance to clinical workflows. The aim was to include surfaces that may serve as potential reservoirs for microorganisms in routine clinical practice and that are simultaneously relevant for transmission between humans, animals, and the environment. This targeted selection enabled a focused assessment of hygienically critical contact surfaces.

The samples were taken as impressions smears (DIN 10113-1) with agar plates provided by Laboklin GmbH & Co. KG (Bad Kissingen, Germany). The samples were collected directly from the respective surfaces by applying even pressure with the agar surface onto the area to be examined. After sampling, the agar plates were stored under refrigerated conditions (+2 °C to +8 °C) within the clinic during the day in order to minimize uncontrolled microbial growth prior to shipment. In the evening of the same day, the plates were sealed airtight with Parafilm, packaged for protection from impact, and placed into insulated Styrofoam boxes with cool packs. The subsequent shipment to the microbiological laboratory of Laboklin GmbH & Co. KG was carried out via Laboklin’s internal courier service from the Berlin site. Transport of the samples was conducted in compliance with the cooling conditions prescribed for microbiological specimens, within a temperature range of +2 °C to +8 °C.

The first sampling period was performed in November 2021. The surfaces were sampled before and after cleaning and disinfection, regardless of macroscopic contamination. The interval that passed between cleaning and disinfection of the surfaces and sample collection varied according to the specific material properties and the practical implementation of the cleaning schedule. Sampling only took place after the measures were fully completed as required and after the surfaces had dried sufficiently.

After the first sampling period, the cleaning schedule was reviewed and revised. Furthermore, additional surfaces were included in the examination scheme. The changes in the examination scheme were implemented in January 2022. Furthermore, the janitors and the clinical staff were informed about the cleaning deficiencies detected in sampling period 1, such as insufficient contact time and concentration of the effective agent, use of the same cleaning cloth for the same areas, skipping important surfaces such as walls or shelves, and stocks), and use of a dirty scrubber dryer machine. Both cleaning schedules are available in Supplementary File S1.

A second sampling period was performed in February 2022. Samples were taken both before and after cleaning in this instance as well. In sampling period 1 and 2, 76 surfaces were sampled, so that in total, 152 samples were examined per sampling period. The third sampling period in June 2022 focused on 21 critical surfaces out of the originally tested 76 areas, leading to 42 samples in total.

Within sampling period 2, few additional surfaces were sampled while few surfaces were excluded from the sampling. Sampling period 3 focused on critical surfaces exclusively. Critical surfaces were defined as surfaces in close contact with the patient or as surfaces that are often touched but easily forgotten in the cleaning process. All in all, 17 areas were sampled three times, 45 areas were checked twice, and 29 areas were checked only once. Details on the sampled surfaces are given in Supplementary File S2.

### 2.2. Samples from Wounds

To identify hospital-acquired surgical site infections with *Acinetobacter* spp., samples of infected wounds were collected. The samples were collected routinely to identify the pathogens involved and to select the appropriate antibiotic based on antimicrobial susceptibility testing. Upon admission of their horses, owners consented to the anonymized use of their horses’ data for scientific purposes. Included in the analysis were exclusively swabs from infected surgical sites either during the initial hospitalization or a readmission to the hospital due to surgical site infection.

The samples were collected by clinical staff, such as veterinarians or veterinary nurses, between October 2021 and June 2022, so that the wound sample collection started one month prior to the first surface sampling period.

### 2.3. Microbiological Examinations

After arrival at the microbiology laboratory of Laboklin GmbH & Co. KG (Bad Kissingen, Germany), agar plates (RODAC contact plates for surface examination and Columbia blood and Endo agar for wound swabs) were inoculated and incubated at +37 °C with macroscopic examination after 24 and additionally after 48 h under aerobic conditions. Additionally, the swabs originating from wounds were incubated in enrichment broth (trypticase soy broth, Becton Dickinson, Heidelberg, Germany) for 16–24 h at 37 °C with subsequent subculture on Columbia blood and Endo agar (Becton Dickinson, Heidelberg, Germany) for 16–24 h at 37 °C and evaluation. Matrix-assisted laser desorption/ionization time-of-flight mass spectrometry (MALDI-TOF-MS, Bruker, Ettlingen, Germany) was used to identify the isolated bacteria. This method allowed for the unambiguous identification of each bacterial species using the current version for the time (MALDI Biotyper Reference Library MBT Compass Library Revision L (2020) 9607 MSP covering 3239 species/entries (9607 MSP); beginning in December 2021, MBT Compass Library Revision H (2021) covering 3893 species/entries (10833 MSP), Bruker Daltonics, Bremen, Germany). According to the software used (MALDI Biotyper Reference Library), genus and species determination was provided when the score was 2 or higher, genus was applied when the score was within 1.7 and 2, and results of 1.7 and below were considered to be unreliable. Confirmation by molecular methods was not carried out, and thus, there was no classification done concerning independent or clonal origin of the isolates.

In sampling period 1, antibiograms of the isolates were also generated by using broth microdilution (CLSI-vet, MIC-based testing, human-derived breakpoints, as there are currently no breakpoints for samples from horses available, with reporting as susceptible, intermediate, or resistant according to the CLSI database). Additionally, internal controls and biannual external controls were implemented. Minimum inhibitory concentrations (MIC) were categorized as sensitive (S), intermediate (I), or resistant (R; according to CLSI M100 Edition 36, 2006). These categories were used to calculate resistance proportions for isolated *Acinetobacter* spp. across several active substances. For presentation, antibiotics used in the clinic were selected. These include amoxicillin, ampicillin, ceftiofur, cefquinome, doxycycline, enrofloxacin, gentamicin, marbofloxacin, penicillin, and tetracycline, as well as trimethoprim and sulfamethoxazole in combination. The panel is not specific for *Acinetobacter* spp. but is designed to aid treatment of all bacterial isolates found in equine practice and thus does not consider intrinsic resistances.

A statistical analysis was omitted, since only few *Acinetobacter* spp. were detected, and there was a reduction in sample size.

## 3. Results

### 3.1. Prevalence of Acinetobacter spp.

During the first sampling period, *Acinetobacter* spp. was detected in at least one sample in each of the different areas. This included detection in both environmental areas, such as the floors and the walls, and in equipment directly in contact with the horses, such as endotracheal tubes.

After revising the cleaning schedule, fewer surfaces tested positive for *Acinetobacter* spp. in sampling period 2, but surfaces in critical areas such as the surgical theatre still tested positive for *Acinetobacter* spp. The reduced detection rate remained consistent months after the implementation of the revised cleaning schedule in sampling period 3. In sampling period 1, 32.9% (25/76) of samples tested positive for *Acinetobacter* spp. before cleaning and disinfection compared to 26.3% (20/76) afterwards. In sampling periods 2 and 3, positivity rates were 14.5% (11/76) and 14.2% (3/21) before cleaning and disinfection and 2.6% (2/76) and 9.5% (3/21) afterwards.

An overview of the surfaces and *Acinetobacter* spp. detection is provided in the three tables below (Table 1, Table 2 and Table 3).

During the study period, samples of surgical site infections were submitted for bacterial culture. Of these, 38 were regarded as nosocomial infections. *Acinetobacter* spp. was not detected in any of these 38 samples.

### 3.2. Resistance Profiles

**Figure 1:**
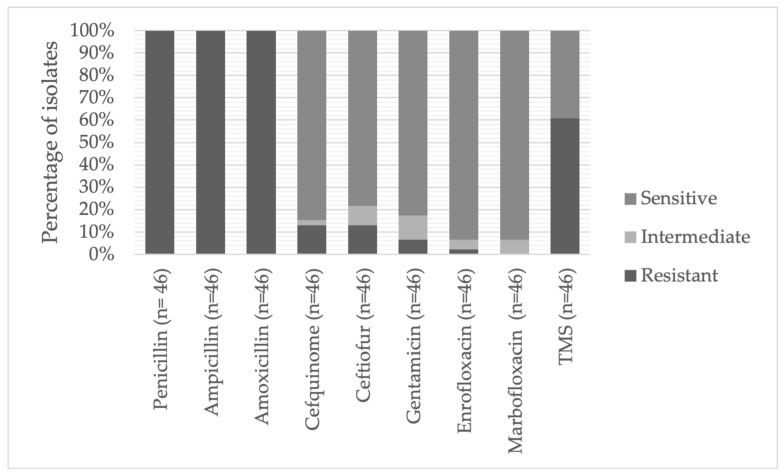
Resistance rates of the *Acinetobacter* isolates against commonly used antibiotics in equine medicine. TMS: trimethoprim and sulfamethoxazol.

Most isolates were highly resistant against beta-lactam antibiotics such as penicillin, against aminopenicillins such as ampicillin (97.9%) and amoxicillin (100%), and against macrolide antibiotics (100% resistance) such as azithromycin, erythromycin, spiramycin, and clarithromycin, as well as against the lincosamide antibiotic clindamycin. The isolates were mostly sensitive to the fluoroquinolones enrofloxacin (2.2% resistance) and marbofloxacin (0.0% resistance), as well as aminoglycosides (gentamicin, 6.5% resistance; kanamycin, 8.7% resistance). Figure 1 shows resistance data for commonly used antibiotics in equine medicine.

The *Acinetobacter baumannii* isolated from the manger in the isolation stable during sampling period 1 was sensitive to cefquinome, ceftiofur, gentamicin, enrofloxacin, tetracycline, and doxycycline when only looking at the commonly used antibiotics in equine medicine.

## 4. Discussion

Environmental sampling of our equine hospital yielded recovery of non-*baumannii Acinetobacter* spp., including *A. lwoffii*, *A. johnsonii*, *A. pseudolwoffii*, and *A. beijerinckii*, with only one single detection of *A. baumannii*. Of the genus *Acinetobacter*, *A. baumannii* is the most common species associated with nosocomial infections, even though involvement of other Acinetobacter species in hospital-acquired infections has been reported previously, especially in humans [26,27,28,29,30,31,32]. The reduction of environmental *Acinetobacter* spp. underlines the effectiveness of the revision of the cleaning schedule and staff training. The effects of training and monitoring were shown in previous studies and are now, in addition to regular reminders, incorporated in the WHO Guidelines on Hand Hygiene in Health Care [31,32,33]. The results also show that clinical facilities might benefit from repeated training, since the percentage of positive samples increased in sampling period 3 after cleaning and disinfection when compared to sampling period 2. Possible effects of season, patient density, case distribution, and staff changes on the recovery rates of *Acinetobacter* spp. cannot be completely excluded. Additionally, it is worrying that some surfaces tested positive for *Acinetobacter* spp. both before and after cleaning and disinfection or only afterwards. This may be attributed to pathogen persistence on the surfaces or by the redistribution of microorganisms during cleaning procedures, which was described during cleaning with detergent wipes [34] or by using contaminated cleaning equipment [35].

In human hospitals, *A. baumannii* has emerged as a major cause of healthcare-associated infections, including ventilator-associated pneumonia, bloodstream infections, and postoperative/wound infections, frequently in immunocompromised or critically ill patients [12,28,36,37]. Also, the descriptions of wound infections, postoperative soft-tissue infections, and urinary tract infections linked to multidrug-resistant *Acinetobacter* spp. in companion animal reports emphasize the therapeutic challenges posed by resistance to multiple antimicrobial classes [16,17,18]. The involvement of *Acinetobacter* spp. in equine infections, such as thrombophlebitis [13,19], respiratory infections [20,21], and neonatal sepsis [22], underlines the susceptibility of equines to this bacterial species. In the present study, *Acinetobacter* spp. was detected in several areas of the equine clinic environment during the examination period, including surfaces frequently contacted by horses, personnel, or equipment. Despite this environmental presence, no surgical site infections associated with *Acinetobacter* spp. were documented during the study period. The limited clinical relevance of *Acinetobacter* spp. in surgical site infections in our clinic during the sampling period indicates that the environmental contamination of the surgery and orthopedic treatment area did not translate into wound infection. The predominance of low-virulence, non-*baumannii Acinetobacter* species in the environment, effective perioperative asepsis, and preoperative preparation of the surgeon may be the reason for this circumstance. In addition, the absence of *Acinetobacter* spp. in surgical site infections of clean surgical wounds suggests that established hygiene and biosecurity practices during the postoperative care are effective and prevent environmental microorganisms from gaining access to surgical sites. Furthermore, these findings emphasize the distinction between mere environmental presence and true nosocomial pathogenicity, highlighting that environmental detection alone should not be equated with clinical relevance. The presence of *Acinetobacter* spp. in infections other than surgical site infections was not evaluated with this study; therefore, it may be possible that *Acinetobacter* spp. played a role in infections other than those of the surgical site.

The revision of the cleaning and disinfection protocol resulted in a descriptive decrease in the detection of *Acinetobacter* spp. This further limited the risk of contracting a surgical site infection with *Acinetobacter* spp. during the study period. However, it must be kept in mind that the sample size decreased during the study, since sampling period 3 only focused on sensitive surfaces, and therefore, only 21 surfaces were examined. If all 76 (or more) surfaces were sampled during sampling period 3, the recovery rate of *Acinetobacter* spp., and especially of *A. baumannii*, may have been higher.

*Acinetobacter* spp. commonly exhibits high rates of bacterial resistance, including resistance to aminoglycosides, fluroquinolones, β-lactams, and cephalosporins, as well as carbapenems, with *Acinetobacter baumannii* commonly being the most resistant species [38,39,40,41,42,43,44]. The challenge of multidrug-resistant *Acinetobacter baumannii* in equine medicine is emphasized by isolates from horses—including samples from the urogenital tract and wounds/abscesses—exhibiting high resistance rates against commonly used antibiotics. This also emphasizes the challenges of their multidrug resistance, including resistance against gentamicin, enrofloxacin, marbofloxacin, tetracycline, and trimethoprim/sulfamethoxazole [45], which are commonly used in horses. An important finding of this study is that the environmental *Acinetobacter* spp. in the examined clinical environment were susceptible to several antibiotic classes, including the single *A. baumannii* isolate from the manger in the isolation stable during sampling period 1. This contrasts with the pronounced multidrug resistance and carbapenem resistance increasingly observed in human, companion animal, and equine nosocomial *A. baumannii* isolates [16,17,18,28,45,46]. The absence of highly resistant isolates in this equine setting may reflect differences in antimicrobial selection pressure or species composition within the *Acinetobacter* genus.

The predominance of non-baumannii *Acinetobacter* spp. that are susceptible to a variety of antibiotics in our clinic is in line with findings from a study evaluating non-hospital environmental *Acinetobacter* spp. loads [47], indicating a lower selective pressure for resistance, potentially resulting from stewardship-compliant antimicrobial prescribing. Since the recovered isolates are mostly sensitive to the combination of trimethoprim-sulfamethoxazol, tetracycline, doxycycline, and the fluoroquinolones enrofloxacin and marbofloxacin, as well as the aminoglycoside gentamicin, it should be possible to effectively treat infections with the recovered isolates.

Despite the lack of clinically apparent infections, the detection of *Acinetobacter* spp. in the clinic environment remains noteworthy. Environmental contamination of the clinic, especially the stables, may act as a reservoir for opportunistic pathogens and could contribute to infection risk under certain conditions, for example, untreated pituitary pars intermedia dysfunction in aged horses [48]. Furthermore, *Acinetobacter* spp. epidemiology in humans has shifted over time towards greater antimicrobial resistance and more virulent lineages [49,50] which may also happen in our facility. Continuous surveillance and routine environmental hygiene protocols in equine clinics therefore remain justified, particularly in high-risk units such as surgical theatres and intensive care stables.

When interpreting these study results, the limitations of the study should be taken into account. An insufficient number of isolates were detected on the same surfaces to allow for a meaningful statistical evaluation of differences between the sampling periods. Since there were also no nosocomial surgical site infections caused by Acinetobacter, a statistical analysis was not meaningful in this case either. Therefore, the presented data remain descriptive and should thus be interpreted with sufficient care. Furthermore, the sampled surfaces did not remain precisely the same during the three sampling periods, which does not allow exact comparison between the three sampling periods.

## 5. Conclusions

Overall, these findings indicate that despite environmental persistence, *Acinetobacter* spp. held a low clinical relevance regarding surgical site infections and did not show high rates of resistance in the equine clinic of the Freie Universität Berlin during the study period. Nevertheless, longitudinal surveillance is warranted to assess whether environmental strains in equine clinics acquire resistance traits or pathogenic potential over time. Adherence to antimicrobial stewardship guidelines may help to preserve the antibiotic susceptibility of *Acinetobacter* spp., which highlights the importance of bacteriological analysis and resistance testing.

## Figures and Tables

**Table 1:** Detection of *Acinetobacter* spp. in the surgical area during the three sampling periods.

Sampled Location	Species	1		2		3	
		Before	After	Before	After	Before	After
Ceiling supply unit	*A. johnsonii*	n.d.	n.d.	0	0	0	x
Endotracheal tube	*Acinetobacter* spp.	x	0	n.d.	n.d.	n.d.	n.d.
Floor 1 (induction area)	*Acinetobacter* spp.	x	0	0	0	n.d.	n.d.
Floor 1 (recovery stable)	*Acinetobacter schindleri*	x	0	0	0	n.d.	n.d.
Floor 1 (surgical theatre)	*Acinetobacter* spp.	x	0	0	0	n.d.	n.d.
Floor 2 (recovery stable)	*Acinetobacter* spp.	x	0	0	0	n.d.	n.d.
Floor 2 (surgical theatre)	*A. johnsonii*	0	x	x	0	0	0
Lamp	*A. johnsonii*	0	0	0	0	0	x
Light switch	*A. lwoffii*	0	x	0	0	0	0
Loop (surgical theatre)	*Acinetobacter* spp.	0	0	x	0	n.d.	n.d.
Surgical table	*Acinetobacter* spp.	x	0	n.d.	n.d.	n.d.	n.d.
Wall 1 (surgical theatre)	*A. lwoffii*	x	0	n.d.	n.d.	n.d.	n.d.
Wall 2 (induction area)	*A. lwoffii*	0	0	x	0	n.d.	n.d.

*A.* = Acinetobacter; spp. = species; 1 = sampling period 1, November 2021; 2 = sampling period 2, February 2022; 3 = sampling period 3, June 2022; x = the organism was present on the selected surface in the selected sampling period; 0 = not detected; n.d. = not done—surface was not sampled in the respective sampling period.

**Table 2:** Detection of *Acinetobacter* spp. in the examination area during the three sampling periods.

Sampled Location	Species	1		2		3	
		Before	After	Before	After	Before	After
Cabinet handle (internal medicine)	*A. lwoffii*	0	x	0	0	0	0
Computer keyboard (orthopedics)	*Acinetobacter* spp.	x	0	0	0	n.d.	n.d.
Computer keyboard (orthopedics)	*A. lwoffii*	0	x	0	0	n.d.	n.d.
Computer mouse (internal medicine)	*A. schindleri*	x	0	0	0	0	0
Computer mouse (orthopedics)	*A. lwoffii*	0	x	0	0	n.d.	n.d.
Endoscope 180 cm (internal medicine)	*A. lwoffii*	0	x	0	0	0	0
Floor (orthopedics)	*Acinetobacter* spp.	x	0	0	0	0	0
Floor 1 (internal medicine)	*A. lwoffii*	x	0	0	0	n.d.	n.d.
Floor 1 (internal medicine)	*A. johnsonii*	0	0	0	x	n.d.	n.d.
Floor 1 (internal medicine)	*Acinetobacter* spp.	0	0	x	0	n.d.	n.d.
Floor 2 (internal medicine)	*A. lwoffii*	x	x	0	0	n.d.	n.d.
Mat (endoscopy cart, additional)	*A. lwoffii*	n.d.	n.d.	0	0	x	0
Shelf space (orthopedics)	*A. lwoffii*	0	x	0	0	0	0
Shelf space (orthopedics)	*A. pseudolwoffii*	0	0	0	0	x	0
Stocks frontside (internal medicine)	*A. lwoffii*	x	0	0	0	n.d.	n.d.
Table (orthopedics)	*Acinetobacter* spp.	x	0	0	0	0	0
Ultrasound probe (orthopedics)	*A. lwoffii*	0	x	0	0	0	0
Ultrasound probe (orthopedics)	*Acinetobacter* spp.	x	0	0	0	0	0
Wall 2 (internal medicine)	*A. radioresistens*	0	x	0	0	n.d.	n.d.

*A.* = Acinetobacter; spp. = species; 1 = sampling period 1, November 2021; 2 = sampling period 2, February 2022; 3 = sampling period 3, June 2022; x = the organism was present on the selected surface in the selected sampling period; 0 = not detected; n.d. = not done—surface was not sampled in the respective sampling period.

**Table 3:** Detection of *Acinetobacter* spp. in the stabling area during the three sampling periods.

Sampled Location	Species	1		2		3	
		Before	After	Before	After	Before	After
Floor 1 (intensive care)	*Acinetobacter* spp.	x	x	0	0	0	0
Floor 1 (special hygiene stable)	*A. pseudolwoffii*	0	0	x	0	n.d.	n.d.
Floor 2 (intensive care)	*Acinetobacter* spp.	x	x	0	0	n.d.	n.d.
Floor 2 (intensive care)	*A* *. lwoffii*	0	x	0	0	n.d.	n.d.
Floor 2 (intensive care)	*A. pseudolwoffii*	0	0	x	0	n.d.	n.d.
Floor 2 (special hygiene stable)	*A. pseudolwoffii*	0	0	x	x	n.d.	n.d.
Manger (intensive care)	*Acinetobacter* spp.	0	x	x	0	n.d.	n.d.
Manger (isolation stable)	*A. baumannii*	x	0	0	0	n.d.	n.d.
Manger (isolation stable)	*A. johnsonii*	0	x	0	0	n.d.	n.d.
Manger (special hygiene stable)	*A. pseudolwoffii*	0	0	x	0	n.d.	n.d.
Stable door handle (isolation stable)	*A. johnsonii*	x	0	0	0	n.d.	n.d.
Stable door handle (isolation stable)	*Acinetobacter* spp.	0	x	0	0	n.d.	n.d.
Stable door handle (special hygiene stable)	*A. lwoffii*	0	0	x	0	n.d.	n.d.
Stable door handle (special hygiene stable)	*Acinetobacter* spp.	0	0	0	x	n.d.	n.d.
Wall 1 (isolation stable)	*A. johnsonii*	x	0	n.d.	n.d.	n.d.	n.d.
Wall 1 (special hygiene stable)	*A. bohemicus*	x	0	n.d.	n.d.	n.d.	n.d.
Waterer (intensive care)	*A. johnsonii*	x	x	0	0	0	0
Waterer (intensive care)	*Acinetobacter* spp.	x	0	0	0	0	0
Waterer (intensive care)	*A. pseudolwoffii*	0	0	0	0	0	x
Waterer (isolation stable)	*A. beijerinckii*	0	x	n.d.	n.d.	n.d.	n.d.
Waterer (special hygiene stable)	*A. johnsonii*	0	0	x	0	n.d.	n.d.
Waterer (special hygiene stable)	*A. lwoffii*	x	x	0	0	n.d.	n.d.
Waterer (special hygiene stable)	*A. pseudolwoffii*	0	0	0	x	n.d.	n.d.
Waterer (special hygiene stable)	*A. lwoffii*	x	x	0	0	n.d.	n.d.

*A.* = Acinetobacter; spp. = species; 1 = sampling period 1, November 2021; 2 = sampling period 2, February 2022; 3 = sampling period 3, June 2022; x = the organism was present on the selected surface in the selected sampling period; 0 = not detected; n.d. = not done—surface was not sampled in the respective sampling period.

## Data Availability

The data are available upon request from the authors.

## Supplementary Materials

The following supporting information can be downloaded at: https://www.mdpi.com/article/10.3390/antibiotics15060558/s1, Supplementary File S1: Cleaning Schedules, Supplementary File S2: Sampled Surfaces.
