# Supplementary material for: Molecular Identification and Phenotypic Antimicrobial Resistance of Acinetobacter spp. from an Equine University Clinic in Germany
Source: Antibiotics (Basel). 2026 May 30;15(6):558. doi: 10.3390/antibiotics15060558 (PMC13295674; doi:10.3390/antibiotics15060558)
Supplement: Supplementary file 1 [file antibiotics-15-00558-s001.zip › Supplementary File S1.pdf]

Overview of the external cleaning and disinfection schedule of the equine clinic, divided into different areas, version before January 2022

**Surgical Area**								
**Task**	**Frequency**	**Mon**	**Tue**	**Wed**	**Thu**	**Fri**	**Sat**	**Remarks**
Pre-cleaning, alternating with alkaline/acidic detergent, afterwards disinfection with Hexaquart 0,5 %	6x weekly	x	x	x	x	x	x	All rooms
Intensive cleaning of floors and walls	1x monthly						x	Surgical theatre 1 and 2, preparation, corridor, sterile access area, sterilization area, CT room
Mashine cleaning of the floors with alternating acidic/neutral detergent	1x weekly					x		005, 010
Cleaning and disinfection of stainless steel furniturre including surfaces and sinks	5x weekly	x	x	x	x	x		All rooms (Sinks in the corridor)
Cleaning of interior windows	1x weekly					x		Corridor, 013, CT room, 005

Cleaning of all white cabinets and surfaces	5x weekly	x	x	x	x	x		All rooms
Dry cleaning of all monitors (6)	1x weekly					x		All rooms
Cleaning and disinfection with Hexaquart 0,5 % wipes								
All door handles	5x weekly	x	x	x	x	x		All rooms
All doors including windows and frames	1x weekly				x			All rooms including 005
All door handles of white cabinets	1x weekly	x						Corridor 013
Stainless steel cabinets	1x weekly		x					Surgical theatre 1+2 (015, 018), preparation, 008 and 019, sterilization area 022
Cleaning of the drains (9 units) and drainage channels (5 units), no disinfection required (including thorough cleaning/scrubbing of the cover grates)	1x weekly				x			Rinse down, open the drain, and clean it from the inside
Rinse and wipe coated walls (also as needed outside the 3-day interval in the event of contamination)	3x weekly	x		x		x		Surgical theatre 1+2 (014, 017), preparation 008 and 019
Walls of the recovery boxes, including doors and the swivel wall-mounted storage area.	1x weekly						x	Surgical theatre 1+2 (008, 019)
Ceiling supply units (CSUs) and ceiling lights	Every 3 months	January April July October						Surgical theatre 1+2 (008, 019)
Skirting boards	Every 3 months	January April July October						All rooms
Top of cabinets	Every 3 months	January April July October						Preparation 008 and 019

Surface of the storage area for the washer-disinfector (WD) and large & small sterilizers	1x weekly			x				Sterilisation 022
Exterior surfaces of stainless steel cabinets	1x weekly		x					Sterilisation 022
Towel, soap, and disinfectant dispensers	1x weekly				x			All rooms

**Treatment areas**								
**Task**	**Frequency**	**Mon**	**Tue**	**Wed**	**Thu**	**Fri**	**Sat**	**Remarks**
Machine cleaning of floors and disinfection with 0.5% Hexaquart	4x weekly	x	x		x	x		All rooms
Machine cleaning of floors with neutral detergent	1x weekly			x				All rooms
Cleaning and disinfection der Edelstahlarmaturen and door handles mit Hexaquart Reinigungstüchern 0,5 %	5x weekly	x	x	x	x	x		All rooms
Cleaning and disinfection of all stainless-steel furniture including the surfaces and sinks	5x weekly	x	x	x	x	x		All rooms
Wipe coated walls (more frequently in case of heavy contamination)	2x weekly		x		x			33, 34, 35, 36, 37, 53, 54
All doors (including glass panels)	1x weekly			x				All rooms
Cleaning of the drains and drainage channels, no disinfection required	1x weekly		x					All rooms

**Further rooms**								
**Task**	**Frequency**	**Mon**	**Tue**	**Wed**	**Thu**	**Fri**	**Sat**	**Remarks**
Floor cleaning and surface cleaning	1x weekly	x						31, 40, 41
Floor cleaning including clearing the area	1x weekly			x				32, 49, 52
Full-surface cleaning of the glass on the exterior doors and transom windows in the recovery stables	1x monthly							Every second week of the month

Overview of the external cleaning and disinfection schedule of the equine clinic, divided into different areas, version after January 2022

**Surgical Area**								
**Task**	**Frequency**	**Mon**	**Tue**	**Wed**	**Thu**	**Fri**	**Sat**	**Remarks**
Rough cleaning, alternating alkaline and acidic cleaning, followed by disinfection with 0.5% Hexaquart	6x weekly	x	x	x	x	x	x	All rooms
Intensive cleaning of floors and walls	1x monthly						x	Surgical theatre 1 + 2, preparation 1+2, corridor, sterilization area room
Machine cleaning of floors using an acidic cleaner and a neutral cleaner on a weekly alternating basis	1x weekly					x		005, 010
Cleaning and disinfection of all stainless steel furniture, including surfaces and sinks (2 units)	5x weekly	x	x	x	x	x		All rooms (Sinks in the corridor)
Cleaning of the interior windows	1x weekly					x		Corridor 013, CT room 005
Cleaning of all white cabinets and surfaces	5x weekly	x	x	x	x	x		All rooms
Dry cleaning of all monitors/screens (6 units)	1x weekly					x		All rooms
Cleaning and disinfection with Hexaquart 0,5 % wipes								
All door handles, doorknobs, light switches, keyboards, computer mice, lighting control panels, and trash bin lids	5x weekly	x	x	x	x	x		All rooms
All doors (including glass panels and frames)	1x weekly				x			All rooms including 005

All door handles on white cabinets	1x weekly	x						Corridor 013
Stainless steel cabinets	1x weekly		x					Surgical theatre 1+2 (015, 018), Preparation 008 and 019, Sterilisation area 022
Cleaning of the drains (9 units) and drainage channels (5 units), no disinfection required (including thorough cleaning/scrubbing of the cover grates)	1x weekly				x			ausspritzen, Gulli öffnen and von innen reinigen
Spray down and wipe coated walls (also more frerquent in case of contamination)	3x weekly	x		x		x		Surgical theatre 1+2 (014, 017), Preparation 008 and 019
Walls of the recovery stables, including doors and the swivel wall-mounted storage area	1x weekly						x	Surgical theatre 1+2 (008, 019)
Ceiling supply units (CSUs) and ceiling lights	5x weekly	x	x	x	x	x		Surgical theatre 1 + 2 (008,019)
Skirting boards	every 3 months	Januars<br>April<br>July<br>October						All rooms
Top surfaces of cabinets								Preparation 008 and 019
Surface of the storage area for the washer-disinfector (WD) and large & small sterilizers	1x weekly			x				Sterilisation 022
Exterior surfaces of stainless steel cabinets	1x weekly		x					Sterilisation 022
Towel, soap, and disinfectant dispensers	5x weekly	x	x	x	x	x		All rooms

**Treatment areas: Orthopaedics and Internal Medicine**								
**Task**	**Frequency**	**Mon**	**Tue**	**Wed**	**Thu**	**Fri**	**Sat**	**Remarks**
Machine cleaning of floors and disinfection with 0.5% Hexaquart, plus rough cleaning of the examination stocks	4x weekly	x	x		x	x		All rooms
Machine cleaning of floors with neutral cleaner	1x weekly			x				All rooms
Cleaning and disinfection of stainless steel fittings, door handles, doorknobs, light switches, storage surfaces, keyboards, computer mouse, mobile tables including table legs and wheels, mobile plastic cabinet including handles, and trash bin lids using 0.5% Hexaquart cleaning wipes	5x weekly	x	x	x	x	x		All rooms
Cleaning and disinfection of all stainless steel furniture, including surfaces, washbasins, and the handles of disinfectant/soap dispensers, using 0.5% Hexaquart cleaning wipes	5x weekly	x	x	x	x	x		All rooms
Wipe coated walls (more frequently in case of heavy contamination)	2x weekly		x		x			33, 34, 35, 36, 37, 53, 54
All doors (including glass panels)	1x weekly			x				All rooms
Cleaning of the drains and drainage channels, no disinfection required	1x weekly		x					All rooms

Doors	January, April, July, October							All rooms
Intensive cleaning of the examination stocks	1 x mo	x						All rooms

**Further rooms**								
**Task**	**Frequency**	**Mon**	**Tue**	**Wed**	**Thu**	**Fri**	**Sat**	**Remarks**
Floor cleaning and surface cleaning	1x weekly	x						31, 40, 41
Floor cleaning including clearing the area	1x weekly			x				32, 49, 52
Full-surface cleaning of the glass on the exterior doors and transom windows in the recovery rooms	1x monthly							2nd week of the month

**Stable 3.1, 3.2**								
**Task**	**Frequency**	**Mon**	**Tue**	**Wed**	**Thu**	**Fri**	**Sat**	**Remarks**
Machine cleaning of floors and disinfection with 0.5% Hexaquart, plus cleaning/disinfection of the examination stocks	4x weekly	x	x		x	x		Examination room + adjacent computer room
Machine cleaning of floors with neutral cleaner	1x weekly			x				Examination room + adjacent computer room
Cleaning and disinfection of stainless steel fittings, storage surfaces, keyboards, computer mouse, mobile tables including table legs and wheels, mobile plastic cabinet including handles, and trash bin lids using 0.5% Hexaquart cleaning wipes	5x weekly	x	x	x	x	x		Examination room + adjacent computer room
Door handles (excluding stable door handles) and light switches	5x weekly	x	x	x	x	x		Stable building
Cleaning and disinfection of all stainless steel furniture, including surfaces, washbasins, and the handles of disinfectant/soap dispensers, using 0.5% Hexaquar	5x weekly	x	x	x	x	x		Examination room + adjacent computer room

Wipe coated walls (more frequently in case of heavy contamination)	2x weekly		x		X			Examination room
Cleaning of all white cabinets and surfaces	5x weekly	X	x	x	x	x		Examination room + adjacent computer room
Cleaning of the drains and drainage channels, no disinfection required	1x weekly		x					All rooms

**Examination area: Isolation**								
**Task**	**Frequency**	**Mon**	**Tue**	**Wed**	**Thu**	**Fri**	**Sat**	**Remarks**
Machine cleaning of floors and disinfection with 2% Hexaquart, plus cleaning/disinfection of the examination stocks	3x weekly	x		x		x		Examination area: Isolation
Cleaning and disinfection of stainless steel fittings, storage surfaces, keyboards, computer mouse, mobile tables including table legs and wheels, mobile plastic cabinet including handles, and trash bin lids using 0.5% Hexaquart cleaning wipes	3x weekly	x		x		x		Examination area: Isolation
Door handles (excluding stable door handles) and light switches	3x weekly	x		x		x		Examination area: Isolation
Cleaning and disinfection of all stainless steel furniture, including surfaces, washbasins, and the handles of disinfectant/soap dispensers, using 0.5% Hexaquart	3x weekly	x		x		x		Examination area: Isolation
Wipe coated walls (more frequently in case of heavy contamination)	2x weekly	x				x		Examination area: Isolation
Cleaning of all white cabinets and surfaces	3x weekly	x		x		x		Examination area: Isolation

Cleaning of the drains and drainage channels, no disinfection required	1x weekly	x						Examination area: Isolation
Cleaning of the drains and drainage channels, no disinfection required	1x weekly	x						All rooms

**Isolation stables**								
**Task**	**Frequency**	**Mon**	**Tue**	**Wed**	**Thu**	**Fri**	**Sat**	**Remarks**
Cleaning of floors and disinfection with 2% Hexaquart, plus cleaning/disinfection of the examination stand	3x weekly	x		x		x		Isolation stables + after discharge of the patient
Cleaning and disinfection of stainless steel fittings, storage surfaces, keyboards, computer mouse, mobile tables including table legs and wheels, mobile plastic cabinet including handles, and trash bin lids using 0.5% Hexaquart cleaning wipes	3x weekly	x		x		x		Isolation stables + after discharge of the patient
Door handles (excluding stable door handles) and light switches	3x weekly	x		x		x		Isolation stables + after discharge of the patient
Cleaning and disinfection of all stainless steel furniture, including surfaces, washbasins, and the handles of disinfectant/soap dispensers, using 0.5% Hexaquart	3x weekly	x		x		x		Isolation stables + after discharge of the patient
Wipe coated walls (more frequently in case of heavy contamination))	2x weekly	x				x		Isolation stables + after discharge of the patient
Cleaning of all cabinets and surfaces	3x weekly	x		x		x		Isolation stables + after discharge of the patient

Cleaning of the drains and drainage channels, no disinfection required	1x weekly	x						Isolation stables + after discharge of the patient
